# Nanomaterial-based delivery vehicles for therapeutic cancer vaccine development

**DOI:** 10.20892/j.issn.2095-3941.2021.0004

**Published:** 2021-06-15

**Authors:** Jie Liang, Xiao Zhao

**Affiliations:** 1 CAS Key Laboratory for Biomedical Effects of Nanomaterials and Nanosafety & CAS Center for Excellence in Nanoscience, National Center for Nanoscience and Technology of China, Beijing 100190, China; 2 University of Chinese Academy of Sciences, Beijing 100049, China

**Keywords:** Nanomaterial-based delivery vehicles, bioinformatic prediction, neoantigen, personalized vaccines, tumor immunotherapy

## Abstract

Nanomaterial-based delivery vehicles such as lipid-based, polymer-based, inorganics-based, and bio-inspired vehicles often carry distinct and attractive advantages in the development of therapeutic cancer vaccines. Based on various delivery vehicles, specifically designed nanomaterials-based vaccines are highly advantageous in boosting therapeutic and prophylactic antitumor immunities. Specifically, therapeutic vaccines featuring unique properties have made major contributions to the enhancement of antigen immunogenicity, encapsulation efficiency, biocompatibility, and stability, as well as promoting antigen cross-presentation and specific CD8^+^ T cell responses. However, for clinical applications, tumor-associated antigen-derived vaccines could be an obstacle, involving immune tolerance and deficiency of tumor specificities, in achieving maximum therapeutic indices. However, when using bioinformatics predictions with emerging innovations of *in silico* tools, neoantigen-based therapeutic vaccines might become potent personalized vaccines for tumor treatments. In this review, we summarize the development of preclinical therapeutic cancer vaccines and the advancements of nanomaterial-based delivery vehicles for cancer immunotherapies, which provide the basis for a personalized vaccine delivery platform. Moreover, we review the existing challenges and future perspectives of nanomaterial-based personalized vaccines for novel tumor immunotherapies.

## Introduction

As a potential immunotherapeutic approach, cancer vaccines are attractive as enhancements for tumor therapy. Dating back to 1891, William Coley discovered a mixed bacterial vaccine, also named Coley’s toxin, which resulted in inconsistent therapeutic effects when treating cancer patients^1^. With the advent of immunotherapy, bacillus Calmette-Guérin was first used by Morales for the treatment of noninvasive bladder cancer in 1976^2^. Early studies showed the capability of bacterial pathogens to induce nonspecific immune responses, but attempts to treat cancer with effective vaccines was not successful because of the inability to recognize tumor antigens. In parallel with the use of bacterial vaccines, dendritic cells (DCs) were discovered in 1973^3^; vaccination showed their antigen-presenting potential in achieving a powerful stimulation to the immune system. Subsequently, the first human cancer antigen, melanoma-associated antigen 1, was recognized in 1991^4^, and genetically modified whole cell tumor vaccines with the secretion of granulocyte-macrophage colony-stimulating factor were shown to elicit a specific CD8^+^ T cell response in 1993^5^. The next major advance, involving the DC-based prostate cancer vaccine-Sipuleucel-T, was approved by the U.S. Food and Drug Administration (FDA) in 2010^6^. Therapeutic cancer vaccines targeting tumor-associated antigens (TAAs) or tumor-specific antigens (TSAs) have been tested in numerous clinical studies over the past decade^7–9^. For example, IMA90^10^ and KRM20^11^, 2 multi-peptide vaccines consisting of TAAs, were tested in a phase III trial in 2012 and a phase I trial in 2015, respectively. More recently, cancer neoantigen-based vaccines have augmented the antitumor immune response, such as the DC-based vaccine presenting neoantigens for the treatment of human melanomas in 2015^12^, RNA-based and multipeptide-based neoantigen vaccines for melanoma in 2017^13,14^, and the phase Ib study of neoantigen vaccines designed for glioblastoma in 2019^15^. However, the present clinical results have shown challenges in predicting tumor neoantigens because of complex technologies, time-consuming processes, and high expenses. Personalized cancer vaccines, although in the beginning stages, have nonetheless used the repertoire of the immune system to develop preclinical anticancer activities^16^.

Anticancer immunity consists of 3 main phenotypes: immune-desert, immune-excluded, and inflamed phenotypes^17^, which are subject to several key steps, including the release of cancer-cell antigens, antigen presentation, CD8^+^ T cell priming and activation, trafficking and infiltration of CD8^+^ T cells into tumors, and ultimately recognizing and killing cancer cells using CD8^+^ T cells. With the underlying obstructions of the immune system to eradicate cancer, each step can be targeted to address these challenges using multiple therapeutic cancer vaccines, which contribute to triggering the antigen-specific CD8^+^ T cell response^18^. Likewise, the current advancement of therapeutic cancer vaccines can target prior steps of tumor antigen processing and presentation, and can also increase the potency of efficient CD8^+^ T cells against cancer, so neoantigen-based vaccines will especially become a vital addition to facilitate immune evasion in complex heterogeneous tumor microenvironments (TMEs)^19^.

However, new and frequent mutations and genetic instabilities result in a negative impact on the identification of corresponding neoantigens^20^, even with high variability in a relapsing tumor, which in turn, demonstrates the challenge in developing personal and effective cancer vaccines^21,22^. Although personalized cancer vaccines are difficult to develop, they increase the magnitude of adaptive immune efficiencies and vaccine immunogenicities^23^. To overcome the limited pool of immune cells defending tumor invasion, qualitatively new approaches to screen individual-specific immunogens and efficiently deliver these specific antigens to antigen-presenting cells (APCs)^24^ are urgently needed to induce powerful immunities to resist tumor growth. In this regard, the sequence of neoantigenic peptides can be predicted by whole-exome sequencing technology and by prediction algorithms for antigen affinities with major histocompatibility complex (MHC) class I molecules^25,26^. In this case, neoantigen-based cancer vaccines are specifically recognized as foreign by activating immune cells, which cannot be excluded and eliminated rapidly in such immunotolerant TMEs^27^. Importantly, delivery vehicles have been widely utilized for cancer vaccines over the past 30 years, because they improve the stability of antigens, the intensities of immune responses, and the safety of vaccines^28^. In this regard, nanomaterial-based delivery systems designed for neoantigen vaccines offer key design advantages such as controlling the loading and release kinetics of antigens, and protecting the immune cargos from degradation before the generation of antigen-specific immune responses^29^. Consequently, the construction of potent immunomodulatory cancer vaccines accelerated by various vehicles and modern prediction techniques have resulted in unprecedented specificities. In this review, we summarize and clarify different HLA-presented tumor antigens, and also review the use of distinctive delivery vehicles associated with potential benefits and disadvantages, to guide the advanced development of nanomaterials-based strategies for therapeutic cancer vaccines.

## Tumor antigens

The nomenclature of antigens presented on tumor cells has a long history. To optimize these antigens generally involves HLA ligands, which might be qualified as tumor-associated to be expressed on general tumor cells or on remaining tumor-specific cells^30^. Because tumor-specific neoantigens are usually rejected by malignant cells, they are specifically designed for cancer vaccines^31^.

### TAAs

TAAs are autologous proteins that are aberrantly expressed on malignant cells, and may display mutations, misfoldings, and degradation or proteolytical cleavages^32^. Because TAAs can be recognized and subsequently trigger immune responses to attack tumor cells by activating lymphocytes, TAA-targeted cancer vaccines have attracted considerable interest in the field of vaccine design^33^. Typical classes of TAAs include overexpressed antigens in cancer cells^16,34^, such as human epidermal growth factor receptor 2 (HER2), mucin-1 protein (MUC1), survivin and human telomerase reverse transcriptase (hTERT) plus cancer/germline antigens in germline cells, such as New York esophageal cancer antigen-1 (NY-ESO-1), the melanoma-associated antigen (MAGE) family and X antigen family member-1b (XAGE-1b), lineage-specific antigens in specific cells such as tyrosinase, glycoprotein 100 (gp100), melanoma antigen recognized by T cell-1 (MART-1), and prostate-specific antigen (PSA). For TAA-directed therapeutic cancer vaccines, salient clinical trials have been conducted in recent years (**Table 1**). Unfortunately, TAA-derived cancer vaccines were disappointing in clinical trials because they lacked specificity towards tumor cells, and to some extent, induced immunological tolerance^35^. Moreover, TAAs faced the challenge of low affinity to CD8^+^ T cells when there was insufficient expression below the threshold of naive T cell recognition^36^. TAA-directed cancer vaccines therefore need to be developed to precisely conform to the natural recognition and subsequent presentation to effector T cells.

**Table 1:** Clinical trials of TAA-directed cancer vaccines

TAAs	Disease	Vaccine intervention	Trial number	Phase	Status	Locations
HER2	Breast cancer	AdHER2/neu dendritic cell vaccine	NCT01730118	Phase 1	Completed	National Institutes of Health Clinical Center
MUC1	Lung carcinoma	MUC1 Peptide-Poly-ICLC Vaccine	NCT03300817	Phase 1	Recruiting	Mayo Clinic in Rochester/University of Pittsburgh Cancer Institute
Survivin	Recurrent epithelial ovarian cancer	DC-006 vaccine	NCT01334047	Phase 1/2	Terminated	Oslo University Hospital-Norwegian Radium Hospital
hTERT	Metastatic prostate cancer	hTERT mRNA DC	NCT01153113	Phase 1/2	Withdrawn	University of Florida
NY-ESO-1	Prostate cancer/bladder cancer/non-small cell lung cancer	NY-ESO-1 plasmid DNA Cancer Vaccine	NCT00199849	Phase 1	Completed	New York Presbyterian Hospital/UT MD Anderson Cancer Center Houston
MAGE-A3	Squamous cell carcinoma of the head and neck	HPV-16 vaccine/MAGE-A3	NCT00257738	Phase 1	Completed	University of Maryland School of Medicine Baltimore
Tyrosinase	Melanoma	Montanide ISA 51	NCT00184067	Phase 2	Terminated	USC/Norris Comprehensive Cancer Center
gp100	Melanoma	gp100 human melanoma peptide vaccine	NCT00001439	Phase 1	Completed	National Cancer Institute
MART-1	Malignant melanoma	CYT004-MelQbG10	NCT00306566	Phase 1/2	Completed	Centre Pluridisciplinaire d’Oncologie & LICR

The identification of TAAs basically focuses on comparison of transcriptomes of normal and malignant tissues, to examine abnormally expressed gene products, with HLA-ligands detected and matched using tandem mass spectrometry (MS/MS)^37^. These methods of next-generation sequencing provide comprehensive sequences and datasets, confident detection of mutations and gene transcription, and high throughput, as well as validation of HLA-ligands with sequence-identical synthetic peptides^38^. However, there are limitations of next-generation sequencing, such as less correlated HLA presentation, uncertain transcript dynamics, and false positive results^39^. MS/MS also has many disadvantages involving limited HLA-ligand coverage, biased sampling and detection, and low throughput with high effort and input^40^.

Although targeting TAAs has had some early clinical trials, to date, the vaccines lacked potency because of issues with their central or peripheral tolerance mechanisms^35^. In addition, although TAA analyses using sequencing and MS/MS techniques showed successful identification, the results were not supported by adequate clinical data^41^. Thus, more attention should be directed towards developing tumor-specific and potent therapeutic vaccines to favor effective immune responses.

### TSAs

TSAs arise as a result of somatic mutations that are cancer-specific, and which are important for use in cancer vaccines. Given that these antigens are not affected by immune tolerance, therapeutic vaccines targeting TSAs have become potent weapons against malignant tumor by activating the specific immune system^42^. Compared to TAAs, neoantigens or TSAs are very tumor-specific with diverse mutations and are especially suitable for personalized therapeutic vaccines^43^. Recent studies have shown that neoantigen-based vaccines are capable of generating neoantigen-specific CD4^+^ and CD8^+^ T cell responses, helping to develop neoantigen-dominated immunotherapies^44^. Furthermore, the prevalence of neoantigens has led to clinical trials, where the mutational burden decreased for individual cancer treatments^45^. The elementary features of neoantigen-targeted vaccines induced adaptive immune responses including^23^ (1) CD8^+^ T cell-directed specific killing of tumor cells, (2) reinforced and permanent immune memory, and (3) minor immunological tolerance and autoimmunity. Fundamentally, neoantigen vaccines can boost the activity of CD8^+^ and CD4^+^ T cells by including the respective MHC-I-binding and MHC-II-binding neoepitopes^46^. Alternatively, the mixed neoantigen vaccines optimally contain MHC-I-binding and MHC-II-binding neoepitopes to stimulate activation of both CD8^+^ and CD4^+^ T cells. However, neoantigen-targeted vaccines face challenges involving the identification of neoantigens^47^. From hundreds of mutations, neoantigens only account for a fraction of mutations that can bind with MHC molecules with high affinity, and consequently only this small percentage of neoantigens can be presented to CD8^+^ T cells^48^. When considering incomplete translation of mutated regions, limited regions correlating with neoepitopes can be encoded^49^. Moreover, through comprehensive whole-exome or transcriptome sequencing techniques, the subject sequences encoding HLA-binding neoantigen peptides can display false-positive results^50^. Thus, MS/MS can be used as an effective tool to detect HLA-restricted peptides and provide evidence of sequencing results to confirm the ability of neoantigen presentations^51^. Recently, new MHC-binding predictions based on MS data supported the identification and selection of candidate neoantigens that had the potential to be used in personalized vaccines^52,53^.

**Figure 1:**
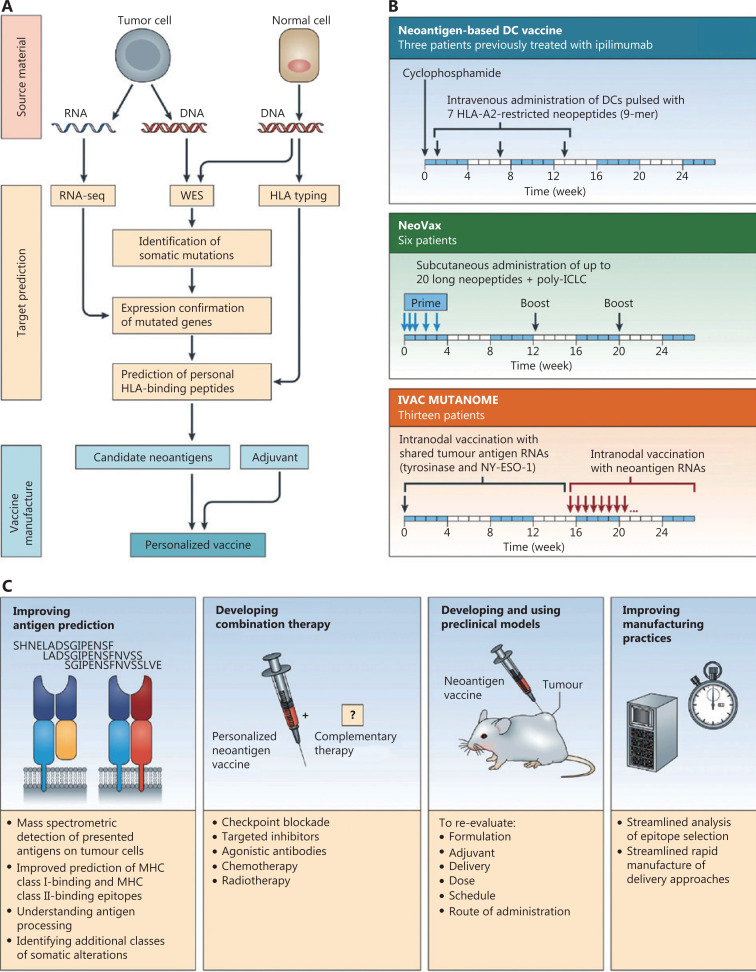
Overview of neoantigen-based therapeutic cancer vaccines by Hu et al.^33^ (A) Neoantigen prediction and vaccine manufacture. (B) Clinical trials of neoantigen-based personalized vaccines. (C) Approaches of enhanced neoantigen-based vaccines.

To increase prediction accuracy, neoantigen prediction algorithms and some recent MHC binding prediction tools have exploited MS training data sets, such as NetMHCpan4 (MHC Class I) and MixMHC2pred (MHC Class II), which accelerated prescreening of MHC binding neoantigens during the prevaccination period^54^. Regularly updated predictors have been developed to use individually calculated decision thresholds and predictive methods to increase prediction specificities and sensitivities^52,55^. Based on common predictive methods among artificial neural networks (ANN), clustering and linear regression (LR) and ANN-based pan-specific algorithms are the optimal predictors, especially NetMHCpan-4.1 (http://www.cbs.dtu.dk/services/NetMHCpan-4.1/) and NetMHCIIpan-4.0 (http://www.cbs.dtu.dk/services/NetMHCIIpan-4.0/), which outperformed respective high-binding efficiencies between predicted antigens and MHC molecules, because of the availability of MS-eluted MHC ligand data^56^. In general, the current neoantigen prediction workflow of neoantigen-based therapeutic cancer vaccines shown in **Figure 1** shares 6 main modules^33,57^: (1) next-generation sequencing of tumor tissue and HLA haplotype acquisition, (2) candidate neoantigen prediction using predictors, (3) neoantigen peptides validation by MS, ELISpot, and fluorescent antibody-labeled MHC tetramers, (4) neoantigen selection with priority, (5) vaccine formulation with putative neoantigens, and (6) preclinical implementation and evaluation of neoantigen-based vaccines. However, personal neoantigen-derived vaccines are limited to eliciting specific CD8^+^ T cell responses because of the lack of tumor-infiltrating lymphocytes into tumor tissues, and the deficient combination of preselected neoepitopes using *in silico* MHC-I and MHC-II binders^58–60^.

Because the current clinical administration of neoantigens involves mixing with adjuvants or cytokines, neoantigens fail to be presented efficiently because of their rapid release. Further complementary vaccine delivery vehicles should be rationally designed to boost effective antigen delivery and immunostimulation.

## Nanomaterial-based delivery vehicles

The TAAs and TSAs for vaccines have shown versatile use in the defense against diverse cancers. Antigen-based cancer vaccines, especially TSA-derived vaccines, involve potential therapeutic strategies with higher immunogenicities^16^. Free antigens are characterized by poor stability, easy degradation, limited immunogenicity, and deficient targeted delivery to APCs. However, current vaccine administration protocols based on delivery systems have increased rapidly to solve these problems^61^. Nanomaterial-based delivery vehicles provide a facile target for a multi-faceted portfolio, including improving the encapsulation efficiency and stability, enhancing immunogenicity, targeting APCs to promote antigen cross-presentation, and co-delivery of the antigen and immunostimulatory adjuvant or nucleic acid with higher transfection efficiency^62,63^.

**Figure 2:**
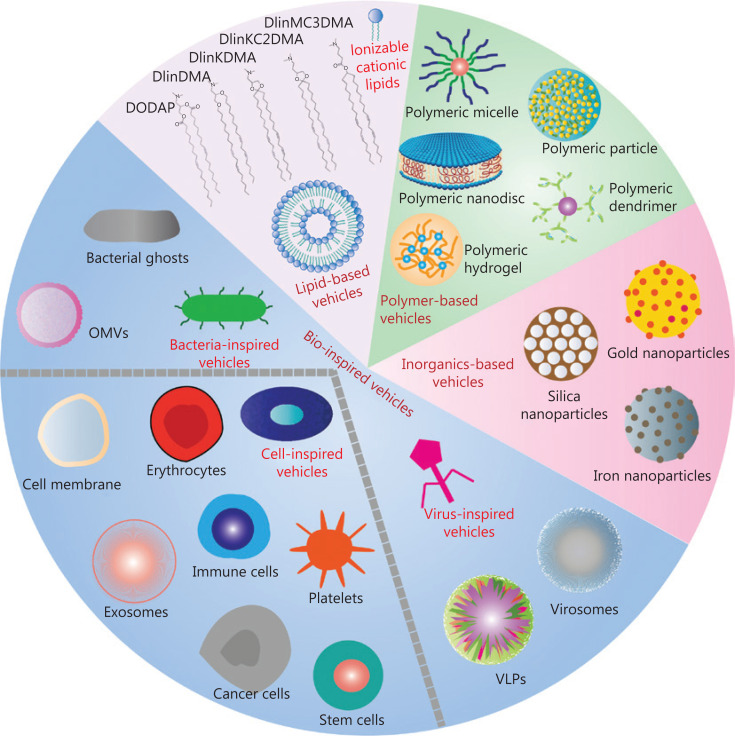
Schematic illustration of nanomaterials-based vaccine delivery vehicles. Different synthetic delivery vehicles such as lipid-based vehicles, polymer-based vehicles, inorganics-based vehicles, and bio-inspired vehicles are used in vaccine construction.

Current potent vaccine platforms evaluated in immunotherapy trials are emerging as a paradigm for TAAs and TSAs delivery^64^. As shown in **Figure 2**, nanomaterials-based delivery vehicles are classified into 2 categories, synthetic delivery vehicles (lipid-based vehicles, polymer-based vehicles, and inorganic-based vehicles) and bio-inspired vehicles. These delivery vehicles can be used for vaccine design as described below.

### Lipid-based vehicles

Lipid-based nanoparticles (LNPs) are promising candidates for vaccine delivery; liposomes are vehicles for clinical applications, which have gained popularity in the design of therapeutic cancer vaccines^65^. With unique spherical vesicles and similarities to the cell membranes, liposomes can encapsulate antigens to make them more stable than free antigens^66^, can easily fuse with cell membranes and preferentially present antigens to APCs, and can enhance immunogenicity and concomitantly trigger adaptive immunity. The first use of liposomes as vaccine delivery systems was approved by Allison and Gregoriadis in 1974, and demonstrated that liposomes served as safe and effective adjuvants in human vaccination protocols^67^. Since that time, liposome use for immunotherapies has become popular, with numerous liposome-based vaccine systems frequently submitted to the FDA for clinical trials^68,69^. In general, liposome-encapsulated antigens can induce both humoral and cellular-mediated immunities, and can protect the antigen from degradation and clearance by the immune system. The delivery vesicle allows antigens to sterically encapsulate into the hydrophilic center or embed into the lipophilic surface. For example, Shahum and Fortin^70^ encapsulated antigens in the internal liposomal aqueous phase or linked them to the liposomal surface. In addition, the use of cationic lipids, neutral lipids, anionic lipids, and PEG-lipids in liposomes has specific structure-functional properties^71^. The advantages of ionizable cationic lipid components (e.g., DODAP, DlinDMA, DlinKDMA, DLinKC2DMA, and DLinMC2DMA^72,73^) are the key elements used to deliver nucleic acid vaccines and bind them to cell membranes for persistent uptake by APCs^74^. Diverse modified cationic lipids elicite greater CD8^+^ and CD4^+^ T cell responses, inducing respective Th-1-based and Th-2-based immune responses, respectively^75^. Neutral lipids are usually used as helper lipids to improve the transfection efficiency of DNA or mRNA vaccines, such as the packaging of 1,2-dioleoyl-sn-glycero-3-phosphoethanolamine (DOPE) and cholesterol to formulate the stable core-shell structure of LNP, which enhanced antigen presentation capabilities^76^. When compared to cationic lipids, anionic lipids are less toxic *in vivo*, with better control, offering a sustained and potent immune response^77^. PEG-modified LNP provides a biocompatible platform for gene transfer, which typically increases the circulation time to extend the stability and effect of vaccines *in vivo*^78,79^. Liposome-based vaccines are also stable and are modified to induce lysosomal escape and antigen cross-presentation^80^. Of particular interest is the use of liposome targeted deliveries with specific ligands or by targeting molecules to the relevant receptors on APCs. For further laboratory-scale and clinical-scale applications of liposomes, the NanoAssembler platform and the NanoAssembler Scale-up platform have been used by Precision NanoSystems (Vancover, Canada) using microfluidic technologies^81–83^.

In addition to liposomes, more advanced LNPs have been developed for vaccine construction, because of lipid-induced higher bioavailability, higher safety, and controlled antigen release. Typical lipids that can self-assemble into LNPs include phosphatidylcholines (DMPC, DPPC, DSPC, DOPC, POPC, and SOPC), phosphatidylglycerols (PG, DMPG, DPPG, DSPG, and DOPG), and other amphiphilic compounds such as phosphatidylethanolamine, phosphatidylserine, and sphingomyelin^66^. Based on a library of lipid-like compounds, the Massachusetts Institute of Technology and Alnylam Pharmaceuticals (Cambridge, MA, USA)^84^ found that efficacious materials such as C12-200 achieved persistent gene silencing and multi-targeted approaches. Kim and colleagues^85^ formulated a small lipid nanoparticle-based nanovaccine platform to display the tumor antigen, ovalbumin (OVA), and combined it with immune checkpoint blockade therapy, which led to an effective antitumor effect. Lipid-assisted nanoparticles also improve the antigen load by using a double emulsion method, and subsequently this method enhances intracellular delivery of antigens to activate immunological responses^86^. To further develop lymph node-targeted delivery of vaccines that result in higher humoral and cellular immune responses, Yu et al.^87^ used melittin-lipid nanoparticles to confirm the ideal LN-target for cancer immunotherapy.

The recent use of LNPs as carriers involves their application in lipid-complexed mRNA vaccines, which increase uptake, extend half-life, and favor efficient delivery of mRNA^13,88^. To address insufficient intracellular mRNA expression and deficient presentation to DCs, a variety of LNP-assisted mRNA vaccines have been designed to deliver mRNA and activate a systemic immune response, such as heterocyclic LNPs to activate immunity *via* the mRNA-mediated STING pathway^89^, mRNA loaded into cationic DOTAP nanoparticles to enhance transfection efficiency^90^, and a nucleoside-modified mRNA-LNP vaccine to efficiently provide potent immune responses^91^. The optimal targets of mRNA vaccination strategies involve efficient antigen delivery into the DC cytosol, as DCs are related to both innate and adaptive immunities. Coolen et al.^92^ modulated the DC-elicited immune response with a lipid-based mRNA delivery system by activating pattern recognition receptors. Another promising approach is remodeling the TME by lipid-complexed mRNA vaccines resulting in the effective delivery of mRNA-encoding immunomodulatory genes. For example, Li^93^ evaluated self-replicating IL-12 RNAs encapsulated in LNPs, which induced long-lasting immune memory, remodeled the TME, and thus primed antitumor immunity. Beyond vaccines against cancer, LNP-encapsulated mRNA vaccines have emerged as an alternative and promising candidate to combat COVID-19 infections^94^. Furthermore, most of these mRNA-based vaccine candidates are currently being tested in clinical trials^95,96^. In conclusion, lipid-based delivery systems validate potential insights toward a comprehensive and productive vector for enhanced vaccine strategies, which may offer effective immune protection against severe diseases such as cancers, viral infections, and pathogen invasions.

### Polymer-based vehicles

Polymer-based vehicles are attracting increasing attention in vaccine development, including antigen encapsulation and protection, conjugations and modifications, and controlled release of cargo with slow degradation^29,97^. Two different types of polymer-based vehicles are used including natural polymer-based and synthetic polymer-based vehicles. Natural polymer-based vehicles are comprised of protein/peptide and glycan nano/microcarriers. In combination with Imiquimod, nab-paclitaxel as an U.S. FDA-approved drug was tested for the treatment of breast cancer cutaneous metastases in a Phase II study^98^. Pancreatic cancer patients treated with nab-paclitaxel plus gemcitabine had a longer survival, which resulted in an improvement in the treatment of this disease^99^. Based on these results, other protein- and peptide-based vehicles such as albumin and self-assembled polypeptide-formulated nanoparticles/micelles can hopefully be utilized for vaccine development. Like natural protein/peptide polymers, chitosan is an amino-polysaccharide derivative of chitin^100^, and cyclodextrins as a family of cyclic oligosaccharides^101^ have attracted interest as a vaccine delivery system, which is nontoxic, consistent with their histocompatibility and biodegradability. Chitosan-based glycan nanoparticles assistant antigens in passing through the epithelial tissue barrier, to prolong the retention time in immunological cells or organs to enhance immunostimulation. Antigens encapsulated into chitosan nanoparticles significantly enhanced the expressions of co-stimulatory signal molecules and pro-inflammatory factors when presented to APCs. Although chitosan has significant advantages as a vaccine delivery system, its low solubility and reticuloendothelial system clearance result in minimal accumulation in tumors. To address this challenge, glycol chitosan nanoparticles provide stimulus-responsive strategies to reject tumors and improve cancer nanomedicine^102^. Cyclodextrin is another popular glycan polymer and 1 of the immunogenic enhancers used in vaccine carrier development. Based on a α-cyclodextrin-fabricated gel system, Qin et al.^101^ developed a versatile gel system for combinatorial tumor therapy, which promoted antigen uptake and DC maturation, and inhibited tumor growth and metastasis. In addition, cyclodextrin carriers promoted MHC I and MHC II binding to enhance the respective proliferation of antigen-specific CD8^+^ T cells and CD4^+^ T cells. For LN-targeted delivery, sulfobutylether-β-cyclodextrin and mannosylated N,N,N-trimethylchitosan DNA vaccines have been developed to enhance DC-targeting and reduce immunosuppression for an efficient antitumor immune response^103^. Collectively, natural polymer-based vehicles are considered as ideal carriers because of their biodegradability, histocompatibility, and mucosal adhesion, but their uneven particle size and weak specificity need to be further refined to facilitate the design of more effective vaccines.

Synthetic polymer-based vehicles are currently widely used to develop numerous vaccines in clinical trials. Owing to their chemical versatility involving covalent or noncovalent binding, synthetic polymer-based vehicles are a potential tool for vaccine delivery. The most extensively used synthetic biodegradable polymers include poly(lactic-co-glycolic acid) (PLGA), polycaprolactone, polylactic acid, polyglycolic acid, polyvinyl alcohol, polyhydroxybutyric acid, polyethyleneimine, and acrylic polymers^104–108^. An example of an U.S. FDA approved polymer is PLGA, which has excellent biocompatibility and biodegradability, sustained and controlled release of antigens, and the ability to be modified to provide specific functions like lysosomal escape^109^. Other synthetic polymers such as polyethylenimine (PEI), a cationic polymer, has been exploited to synthesize linear and branched polymers with different molecular weights^110^. With a positive charge to link nucleic acids through electrostatic interactions, PEI-based nanoparticles are extensively used in gene delivery^111^. Various types of artificial polymers that bind to heparan proteoglycan on the surface of APCs are internalized by cell endocytosis^112^, and the nanoparticles then disintegrate using the proton sponge effect in the acidic environment of lysosomes, thus facilitating cytoplasmic release of loaded substances inside nanoparticles^113^. Polymethylmethacrylate, poly (ethylacrylic acid), poly (propylacrylic acid), and tert-butyl acrylate in acrylic polymers also have several advantages, including a strong immunological adjuvant effect, simple preparation, safety, and biocompatibility^114,115^. Moreover, synthetic polymer-based carriers are usually composed of polymeric micro/nanoparticles, polymeric micelles, dendrimers, nanodiscs, and hydrogels^116–118^. Polymeric micro/nanoparticles are micro/nano-sized colloidal particles that assemble hydrophilic and hydrophobic molecules, stably delivering antigens with good efficacy and with easy entrapment and adsorption^119^. Polymeric micelles are self-assembled spherical structures generated by amphiphilic block copolymers inside aqueous media^120^, which carry a hydrophobic core shell and a hydrophilic outer shell with sizes ranging from 10–100 nm^121^. The internal hydrophobic and surface hydrophilic components of micelles are able to load bioactive molecules with diverse solubility properties. Dendrimers are highly branched spherical carriers consisting of 3 components: a central core, dendritic monomers, and peripheral functional groups^122^. Similar to polymeric micelles, dendrimers are comprised of a hydrophobic core and a hydrophilic surface resulting from polymerization of branching modules^123^. Because of their hyperbranched structures, dendrimers have the characteristics of low viscosity, macromolecular size, and the ability to be chemically modified by covalent or noncovalent binding^124,125^. These synthetic polymer-based vaccines can stimulate and regulate the immune system, and then inflame the tumor microenvironment, and are conducive to inducing systemic antitumor immunity.

Ma et al.^126^ engineered several homogeneous chitosan, PLGA, and PLA-based chassis particles, which displayed antigens on macro/nanoparticles, and which supported excellent antigen delivery and functioned as superior immunostimulants^127^. To further improve immune recognition, Xia et al.^128^ constructed a deformable engineered vaccine using PLGA nanoparticles instead of surfactants, where antigens were assembled on a dynamic flexible emulsion chassis to increase the preventive and therapeutic effects. Compared with bare polymer-based carriers, biodegradable composite materials are being used to engineer vaccines with responsive features, such as autofluorescence, pH sensitivity, and other general physical and chemical properties. PEI-based nanovaccines can be successfully used as powerful tools for visual vaccine delivery and enhanced immunotherapy with autofluorescence^129^. Many pH sensitive polymers (such as PDEA, PEPA, PDPA, PDBA, PC6A, and PC7A) conjugated with antigens, adjuvants, and small molecule agonists, facilitate pH-triggered payload release in acidic tissues, to increase the efficacies of immunological therapies^130^. For better physicochemical properties, PEGylated polymeric nanoparticles and micelles are useful because of their narrow size distribution, homogeneous particle formation, and particular geometry. These nanoparticles co-assemble with subunit antigens to improve the stability of antigens and enhance the cooperativity of material components for specific vaccine performance^131^. In addition, functionalized polymeric vehicles provide new strategies for understanding the metabolism of improved immune effectiveness by virtue of their novel properties with distinctive modifications. Liu and co-workers^132^ recently fabricated F-PEI-based OVA nanoparticles featuring a cross-presentation ability resulting from cytosolic transportation of antigens within dendritic cells, indicating that their fluoropolymers facilitated endosomal escape. For biomaterials-based regulation of signaling pathways, Langer et al.^133^ developed PLGA microparticles encapsulated with a STING agonist to boost the systemic antitumor immune response. Notably, DC-targeted and CD8^+^ T cell-targeted nanovectors contributed to the development of vaccine development and immunization. In a study, Conniot et al.^134^ synthesized mannose-grafted polymeric nanoparticles to target delivery to DCs and subsequent T-cell priming, which potentiated the antitumor immune response. Considering that DCs are used to maximize CD8^+^ T cell activation, synthesized polymers based on artificial APCs aim to directly stimulate CD8^+^ T cells, to trigger stronger T cell proliferation, and to subsequently kill tumor cells^135^. These multifunctional polymeric vehicles therefore hold great promise for vaccine delivery and cancer immunotherapy.

### Inorganic-based vehicles

Vaccines with inorganic-based vehicles support biochemical elaboration elements for superior behavior in cancer immunotherapy. Examples of inorganic-based vehicles include silica, gold, iron oxide, silver, carbon, graphene, selenium, copper oxide, zinc oxide nanoparticles, metal-organic-frameworks (MOFs), and quantum dots^64,136^. Multiple inorganics fabricated vaccines are more preferable in inducing persistent immunostimulatory effects with good *in vivo* stabilities, which are comparable to organic materials^137^. In general, inorganic-based delivery strategies should be used to ensure the enrichment and retention time of antigens in lymphatic circulation, where immune effectiveness is induced and then increased. Diverse representative inorganic nanoparticles are discussed below.

Silica nanoparticles widely studied in cancer therapy are predominantly synthetic amorphous silica based nanoparticles, specifically mesoporous silica nanoparticles (MSNs), which have the advantageous properties of a porous structure, high surface area, tunable surface functionality, and high loading efficiency^138^. MSNs are smart antigen delivery systems that rely on the aforementioned superior properties, which have resulted in their use as vectors^139^. MSNs possess qualities that improve the therapeutic effects, such as protection of vaccine cargo, on-demand release of antigen, antigen targeting transportation, and tumor penetration. In addition, MSNs possess adjuvant behaviors due to the properties of morphology, size, and modified groups^140^. Thus far, a myriad of functionalized silica nanoparticles, acting as adjuvants or carriers, have been used for therapeutic cancer vaccines^141,142^. For example, various types of hollow MSNs have been considered as promising vaccine adjuvants to direct humoral and cellular immune responses and subsequently deprive tumor cells during tumor challenge^143^. To induce greater immune responses, Al-OH-rich silicate adjuvants formulated with 6-coordinate Al-OH groups, using coordination chemistry, induced an effective immunity, which provided new therapeutic options capable of commercial adjuvant development^144^. As vaccine carriers, metal-organic-framework (MOF)-gated MSNs^145^ and mesoporous silica-coated upconversion nanoparticles^146^ accommodated large amounts of antigens using their large pore, niche, and void space, providing the expertise to forgo conventional silica nanoparticle applications. Despite MSNs use as widespread vehicles for cancer vaccinations, mesoporous silica rods (MSRs) recruited APCs in local macroporous scaffolds to induce robust immune activation and antigen-specific adaptive immunity^147^. MSR-MSNs dual-scale vaccines consist of MSNs and MSRs^148^, both of which are internalized by DCs and recruit DCs by virtue of their larger space. This process reverses the immunosuppressive pathway and educates immune cells to combat cancer. Moreover, silica hybrid vaccines such as PEI embedded in MSRs significantly facilitate DC maturation and maximize CD8^+^ T cell stimulation, in contrast to MSR vaccines^139^. As a consequence of the synthetic diversity of silica materials, silica-based vaccines combined with other therapies (e.g., chemotherapy, photothermal therapy, and photodynamic therapy)^147^ overcome the side effects and suppressive efficacies present in tumor pathophysiological processes.

Gold nanoparticles (AuNPs) can serve as an effective antigen delivery system, and can also be used for cancer irradiation therapy; they possess the unique combinatorial therapy of photothermal therapy and immunotherapy. Their inert properties assist them in providing a reliable platform for antigen transportation and medical imaging, as well as providing optical and thermal properties as thermal transducers for near-infrared (NIR)-irradiation tumor therapy^149^. In a previous study, light-activatable polybubbles engineered in this technology were used for antigen delivery vehicles in NIR-responsive cancer treatments^150^. With an extremely small size of 4.5 nm, AuNPs function as vehicles and even adjuvants by activating NLRP3 inflammasomes to induce OVA-specific antibody secretion^151^. The size distribution of AuNPs-based vaccine are therefore used, with different modifications, over a wide range of responses. Zhou et al.^152^ evaluated different-sized AuNPs associated with OVA, and found that 15–80 nm sized vaccines were capable of improving DC homing and inducing CD8^+^ T cell activation. However, the systemic toxicity and decreased specificity of AuNPs limited them in clinical studies. Based on these deficiencies, available surface-modified vaccine delivery vehicles such as PEG-grafted vaccines are the most common in the field of immune engineering. In these studies, PEG increased the stability and safety of AuNPs and enabled tumor accumulation using chemical bonding methodology^153^. Additionally, as imaging agents, AuNPs offer attractive opportunities for medical imaging, including positron emission tomography, magnetic resonance imaging (MRI), and computed tomography (CT), facilitating real-time visualization in tumor diagnoses and treatments^154,155^.

Because of their promising magnetic properties, iron nanoparticles or super paramagnetic iron oxide nanoparticles have also been used as excellent candidates for biomedical diagnoses and imaging. In addition to this property, hyperthermia-based vaccination is also currently used because of the thermal properties^156^. In the field of multifunctional vaccine construction, iron nanoparticles are used as antigen carriers, imaging agents, and heat generation agents with extensive applications in cancer immunotherapy. The clinical administration of iron nanoparticles involves NanoTherm, approved by the U.S. FDA in 2011, which dictates their biological applications; they are accompanied by excellent safety and nontoxicity^157^. Iron nanoparticles accompanied by antigens are therefore synergistic for the treatment of tumors. For early prediction of individual vaccine treatments, iron oxide components are used for DC homing and tracking using MRI. In this study, multifunctional RNA-loaded magnetic liposomes provide a simple procedure to predict the antitumor response^158^, which functions as an early visual tool for vaccine evaluation. With regards to the superior photothermal conversion effect, Guo et al.^159^ fabricated magnetic-responsive nanoagents for precise image-guided immunotherapy, using tumor ablation to release tumor-associated antigens, combined with an immunoadjuvant to elicit robust immunity against tumor growth. Except for the abovementioned autologous vaccine-like nanoagents, the FeO-OVA vaccine efficiently inhibited tumor growth and metastasis, and showed that the FeO-based nanovaccine had better clinical prospects. More importantly, iron oxide nanoparticles were encapsulated in micelles and combined with an immune checkpoint inhibitor such as programmed death-ligand 1 (PD-L1)^160^, which showed the promising result of enhanced immunotherapy during tumor challenge with a high level of effective memory T cells.

### Bio-inspired vehicles

**Figure 3:**
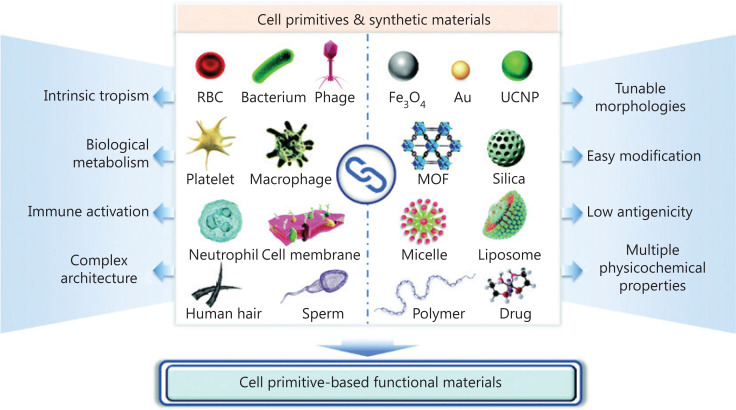
Schematic of the combination of cell primitives and synthetic materials with their functions by Luo et al.^163^

Synthetic delivery vehicles are often excluded by *in vivo* defense mechanisms, weakening the efficacy and safety of vaccine administration, and thus they encounter obstacles for clinical applications. Unlike synthetic delivery vehicles, bio-inspired vehicles increase their potential to associate with physiological elements because they resemble biological systems. They carry the capacities of large drug-loading, antigenicity, adjuvant activity, considerable safety, and targeted delivery^161^. It has been predicted that bio-inspired delivery vehicles will provide a novel opportunity to revolutionize vaccine delivery systems, as well as integrate synthetic materials and bio-inspired agents (**Figure 3**)^162,163^. Notably, 3 kinds of bio-inspired vehicles, including bacteria-inspired, mammalian cell-inspired, and virus-inspired delivery systems, are used for vaccine-based tumor immunotherapy and are described herein in detail.

Bacteria-inspired delivery vehicles such as bacterial ghosts and outer membrane vesicles (OMVs) are predominantly used for therapeutic vaccines^164^. These antigen-loaded vehicles induce safe and potent immune activation, when compared with living bacteria, because of their attenuated pathogenicity and altered immunogenicity. These promising vaccine delivery systems can act as vaccine delivery vehicles, but can also act as adjuvants by virtue of preserved intrinsic immunomodulators such as lipopolysaccharides (LPS) and peptidoglycan^163,165^. Bacterial ghosts derived from Gram-negative bacteria with simple and high dose production procedures feature empty bacterial shells and show structural stability at room temperature. In addition, bacterial ghosts have the safety profile of not being living, but preserve the antigenic units for further vaccine construction^163^. Numerous studies have shown that DNA-loaded bacterial ghosts were effectively internalized and presented to DCs, and amplified direct immune effectiveness in cancer therapy^166^. With bacterial ghost-based DNA delivery vectors, Kudela et al.^167^ used this system to optimize drug delivery and select specific formulations of bacterial ghosts for use in clinical studies. In a similar manner, OMVs are another example of bacteria-inspired delivery vehicles used in advanced vaccine delivery. OMVs are also made from Gram-negative bacteria with certain pathogen-associated molecular patterns, such as LPS, outer membrane proteins, and lipoproteins. Heterogeneous antigens are capable of being presented on the surface of the OMVs through the expression of surface-exposed lipoproteins (e.g., OspA and ClyA)^168^. Beyond the antigen presentation by DCs in modulating the immune response, OMVs can regulate TME reprogramming without side effects^169^. The superior usefulness of OMVs can be used to carry various cargos, such as target antibodies, small interfering RNAs, peptide antigens, and nanoparticles^170^. Altogether, bacteria-inspired delivery vehicles are a promising approach in vaccine development involving antigen delivery and preclinical applications.

Cell-inspired delivery vehicles imitate natural properties of primitive cells, which are used in drug delivery systems with their natural biology of living cells. Based on novel developed nanotechnology, smart cells, especially mammalian cells or cell membranes, integrated with functional nanoparticles, have shown excellent prospects in the field of vaccine fabrication^171^. The main types of cell-inspired delivery vehicles used for tumor immunotherapy are mammalian cell-derived vehicles (e.g., erythrocytes, platelets, immune cells, and stem cells), exosomes, and cell membranes^172,173^. Erythrocytes function as a unique ‘‘don’t eat me’’ signal to hinder phagocytosis and capture certain pathogens to present to APCs. In a recent study, erythrocytes were used to deliver vaccine nanoparticles without additional adjuvants, achieving antigen specific cellular and humoral immune responses^174^. In a similar manner, erythrocyte membranes showed the same features when used to coat antigen-based nanoparticles, where the surface antigens circulated for longer times to elicit a stronger immunity^175^. Platelets and platelet membranes have the advantage of long circulation, damage of immune evasion, self-aggregation, and adhesion at tumor sites for the coating of vaccine nanoparticles^176^. Immune cell-based vaccine vehicles play an important role within a group of immune cells for protecting the host against tumor cells. The types of immune cells include macrophages, neutrophils, DCs, natural killer cells, T cells, and B cells. Because they may trigger undesired immune responses, the immune cells used in vaccine-based tumor immunotherapy are screened. For example, a biomimetic DC-derived nanovaccine fabricated with PLGA nanoparticles by an extrusion method delayed tumor growth and reduced tumor metastasis^177^. In addition, PEI-modified macrophage cell membrane-coated PLGA nanoparticles delivering OVA showed superior therapeutic efficacy with antitumor immune responses^178^. Although stem cells are difficult to collect and maintain their integrity, stem cell-based vaccines are a hopeful therapeutic strategies to treat tumors^179^. Exosome-based bio-inspired vaccine delivery cargoes induced systemic antitumor immunity and long-term immunological memory^180^. In particular, exosomes originating from DCs have the ability to induce antigen-specific T-cell responses for promising vaccine immunization. Additionally, cell membrane-based vaccination promotes immune responses by taking advantage of inherent functions from parent cells^175,181^. In this application, cancer cell membrane-wrapped nanoparticles provided excellent insight into the vaccine delivery and potent immunotherapy^182^. Collectively, such cell-inspired vaccine delivery vehicles, especially with hybrid nanoparticles, generate mimetic vaccine advancement for fighting cancer.

Virus-inspired delivery vehicles are used for vaccine delivery because of their self-replication ability, to transfer specific genes and immunologically escape to target transportation. By combining viral vectors with nanoparticle release, the limitation of nucleic acid cargos could be overcome to increase their versatile applications^172^. Viral vectors such as adenoviruses carry magnetic iron oxide nanoparticles for gene delivery and visual monitoring^183^. However, shortcomings involving safety concerns and restricted loading capacity need to be further addressed. To benefit from viruses and to avoid these concerns, additional vectors such as virus-like particles (VLPs) and virosomes have been introduced^184^. As highly self-assembled particles, VLPs mimic viral capsids and remain as empty shells to preserve antigenicity. They are easy to scale-up at a low cost, and possess natural tropism and targeting capacity with the aid of distinct modifications, which can load antigens for therapeutic vaccine delivery. In addition, the stable architecture of VLPs makes them safer in complex immune systems. Ong et al.^185^ suggested various VLP-based cancer vaccines to be used for the treatment of pancreatic cancer, prostate cancer, breast cancer, and skin cancer, facilitating VLPs as optimal therapeutic vaccine candidates for clinical applications. Similar to VLPs, virosomes also contain a hollow compartment, which allows them to be reconstituted with diverse cargoes. Without the genetic component of viruses, virosomes are commonly produced by influenza virus with low toxicity and empty envelope glycoproteins^186^. These glycoproteins exert adjuvant activity and have the potential of endosomal escape when used for vaccine delivery, showing the substantial advantage of virosomes as delivery vehicles^162^. Because of endosomal escape, virosomes enable DCs to present antigens on the major MHC I molecules to elicit effective cellular immune responses and induce durable tumor suppression. In a previous study, virosomes increased cross-presentation of the encoded immunogen and facilitated DNA vaccine efficacy to an encoded toxic protein, to induce cell death^187^. Thus, both VLPs and virosomes are attractive delivery systems for cancer treatment, representing an important approach for bio-inspired vaccine strategy.

## Current disadvantages and future strategies for further improvement of nanomaterial-based delivery vehicles

Although the use of nanomaterial-based delivery vehicles can facilitate antigen delivery for antitumor therapy with an emphasis on intensified vaccine construction, their clinical translation is difficult because of safety and validity limitations. Regarding synthetic delivery vehicles, lipid-based vehicles present disadvantages, including limited encapsulation efficiency and non-ideal biodistribution, which enhance the aggregation of the liver and spleen cells^29^. Polymer-based vehicles can be limited by toxicity, biodegradability, and impaired immune responses because of chemical modulations^64^. Inorganic-based vehicles increase the risk of solubility and *in vivo* toxicity caused by acute toxic effects^136^. In addition, these synthetic delivery vehicles also limit the fabrication process of composite materials, and adjuvants are needed to improve their use in vaccine immunogenicity. Furthermore, the associated constraints of bio-inspired vehicles include low antigen loading efficiency, superfluous immunogenicity, unpredictable side effects, and even cytotoxicity with susceptible action mechanisms and pharmacokinetic properties^163,166^. Overall, the disadvantages of nanomaterials-based delivery vehicles face the aforementioned obstacles, which involve physical and biological barriers leading to a decrease of clinical vaccine applications, as well as a variety of administration routes that possibly increase the targeting efficiency. This is especially true for nanomaterial-based delivery vehicles, which reflect the tailoring design of these delivery systems to minimize existing risks.

To further improve nanomaterials-based delivery vehicles used as cancer vaccines, more promising designs have the potential to utilize excellent synthesis strategies, such as specific chemical and engineered modifications, bio-responsive moieties and architectures, and incorporation of targeting components^188^. For a notable subset of synthetic delivery vehicles, elaborate material conjugation will improve the physical and chemical features, as well as the immunoregulatory capacities, with remaining adjuvant properties to regulate immune responses and increase the intrinsic safety^68,106^. In particular, more attention should be directed to their beneficial effects, combined with biocompatible and specialized synthetic materials, such as some U.S. FDA approved polymers. Another subclass of vaccine delivery systems is commonly referred to as immunogenic delivery systems, involving bio-inspired vehicles, which can be formulated by genetic manipulation and purification processes to increase their positive antitumor immune responses^172^. Additionally, large-scale preparation enables convenient acquisition of nanomaterials-based delivery vehicles with simple and available preparation procedures, accelerating clinical applications and marketing. Notably, a great deal of cell primitive-based therapeutic delivery vehicles have been developed with increased bioengineering and bioconjugation technologies, which have many advantages, when compared with single delivery systems^164^.

Overall, there are no real insurmountable obstacles to overcome. As an innovative type of advanced vaccine carrier for tumor immunotherapy, hybrid delivery vehicles are currently undergoing testing in many preclinical studies.

## Conclusions and perspectives

The use of nanomaterials-based vaccines has been characterized by a rapidly emerging approach in the advancement of therapeutic cancer vaccines. As discussed in this review, 2 types of tumor antigens and 4 types of nanomaterials-based delivery vehicles have achieved interesting fabrications of cancer vaccines, which is attributed to the unique properties of each profile. Identification of TAAs and TSAs creates a solid foundation for vaccine-based tumor immunotherapy, enabling these vaccines to induce antigen-specific immune responses. However, a multitude of predicted TSAs fails to exist in tumors, and worse still, most predicted neoepitopes are immune privileged when tested for CD4^+^ and CD8^+^ T cell activations. In addition, recent predictors have designed multiple algorithms for predicting MHC-I and MHC-II binders, which elicit CD8^+^ and CD4^+^ T-cell antitumor immunities, respectively. This suggests that the algorithms involving *in silico* tools of MHC-I and MHC-II binders play an important role in personalized immunotherapy to support antitumor immune responses. Although the profundity and accuracy of neoepitope prediction hinders the development of personalized vaccines, immunogenic neoantigens are urgently required for neoantigen-based individual treatments.

This review also suggested that hybrids of different vaccine delivery vehicles are critical for therapeutic purposes, presenting fascinating prospects for future personalized therapeutic vaccines. The functioning of these delivery vehicles, such as for real-time imaging modules, enhancing immunogenicity modules, targeting modules, and environmental responsiveness modules, represents this combinatorial modification, and provides an intriguing strategy for innovative and specific cancer treatments. Among many delivery vehicles, the combination of synthetic delivery vehicles and bio-inspired delivery vehicles has shown reasonable treatment efficacies, because they mimic natural cell characteristics, leading to prominent potentials of bio-friendly vaccine platforms in clinical reality. Nevertheless, this application is still in its preliminary stages, with challenges including coating efficiencies and cost-effectiveness considerations. It is therefore important for researchers to develop a better understanding of biological delivery mechanisms by ameliorating diverse preparation techniques. At the same time, based on computational algorithms, the innovation research of such integrated delivery vehicles should analyze *in vivo* systems to maximize long-term circulation and tumor site accumulation. However, this novel approach requires a large number of samples to verify these algorithms with annotated data. We therefore imagine building optimal delivery vehicles using biological interaction data and computational algorithms to guide future hybrid delivery systems. These delivery vehicles artificially control pharmacokinetic properties of these elaborate therapeutic vaccines with maximum acceptable delivery efficacies to target sites. While currently far from this goal, the physicochemical properties of delivery vehicles interacting with biological environments are being developed for delivery and vaccine design guidance at the sub-organ or subcellular levels.

The broader concept of antitumor immunity used for up-to-date studies will lead to the development of engineered vaccines as adaptive immunomodulators. Effective adaptive immunity has the potential to eliminate tumor cells within a limited time, but excessive inflammation will also promote the progression of tumor growth, owing to complex phenotypes and heterogeneous TMEs. Innate immune training emerges as a novel therapeutic focus, which can promote antitumor activity, eliciting a durable immune response. With the introduced mechanism of trained immunity, nanomaterials-based therapeutic vaccines may be beneficial for cancer treatment involving appropriate reprogramming. These considerations indicate a new design of cancer vaccines, which will accelerate the production of next-generation personalized vaccines to meet the needs of cancer therapy. However, it is essential to transform scientific advances into clinical applications using nanomaterials-based delivery platforms.
